# Flat lens–based subwavelength focusing and scanning enabled by Fourier translation

**DOI:** 10.1515/nanoph-2024-0206

**Published:** 2024-07-11

**Authors:** Xin Zhang, Yanwen Hu, Haolin Lin, Hao Yin, Zhen Li, Shenhe Fu, Zhenqiang Chen

**Affiliations:** College of Physics and Optoelectronic Engineering, 47885 Jinan University, Guangzhou 510632, China; Guangdong Provincial Key Laboratory of Optical Fiber Sensing and Communications, Guangzhou 510632, China; Guangdong Provincial Engineering Research Center of Crystal and Laser Technology, Guangzhou 510632, China

**Keywords:** subwavelength, focusing and scanning, Fourier translation

## Abstract

We demonstrate a technique for flexibly controlling subwavelength focusing and scanning, by using the Fourier translation property of a topology-preserved flat lens. The Fourier transform property of the flat lens enables converting an initial phase shift of light into a spatial displacement of its focus. The flat lens used in the technique exhibits a numerical aperture of 0.7, leading to focusing the incident light to a subwavelength scale. Based on the technique, we realize flexible control of the focal positions with arbitrary incident light, including higher-order structured light. Particularly, the presented platform can generate multifocal spots carrying optical angular momentum, with each focal spot independently controlled by the incident phase shift. This technique results in a scanning area of 10 μm × 10 μm, allowing to realize optical scanning imaging with spatial resolution up to 700 nm. This idea is able to achieve even smaller spatial resolution when using higher-numerical-aperture flat lens and can be extended to integrated scenarios with smaller dimension. The presented technique benefits potential applications such as in scanning imaging, optical manipulation, and laser lithography.

## 1 Introduction

The development of laser scanning technology at the micro-/nanoscale is becoming more and more important as it is widely utilized in various fields, such as optical microscopy imaging [1]–[4], particle trapping and manipulation [5], and sensing [6], [7]. Particularly, the research on controlling subwavelength focusing, including monofocal and multifocal arrays, has aroused much interest, since it can significantly improve scanning efficiency and quality of the scanning images.

There are currently two major principles of laser scanning, namely, the displacement scanning and the computational scanning. The displacement scanning is typically achieved by shifting the illumination source or the sample using a mechanical stage. The target information is, therefore, obtained through the merging of point-by-point scanning images [8]. Confocal microscopy system is a commonly used scanning technique [9]–[11]. Placing the pinhole in a focal plane that is conjugate to the object sample allows the system to collect light from highly focused points [12]. The advantages of this scanning technique are the high resolution [13]–[15] and the ability to reconstruct optical images in three dimensions [16]. To further enhance scanning imaging resolution, super-oscillatory lenses have been currently proposed and realized to achieve a scanning focal spot smaller than the diffraction limit [17]–[19]. However, the generated super-oscillating light spot is accompanied by strong side lobes, which limit the field of view in the imaging system. Furthermore, the structural complexity of the super-oscillatory lenses, together with the intricacy of its control mechanisms, presents significant challenges in practical applications.

By comparison, computational scanning technique, without mechanical movement, mitigates those limitations. For example, the spatial position of the focal spot can be effectively controlled by modulating phase or amplitude of the incident light using a computer-generated hologram [20]–[24]. In this scenario, various algorithms have been exploited to generate segmentation phase holograms, which are successively uploaded onto a spatial light modulator (SLM) for controlling transverse position of the multifocal points [25]–[30]. In addition to the computer-generated holograms, it is also possible to encode phase and amplitude information into various kinds of computer-designed diffractive optical elements including metasurfaces [31], [32], metalens [33]–[45], lens arrays [46], and gratings [47], [48]. Due to diffraction of the elements and the holograms, we can split a single incident beam into multiple focal beams at the same focal plane. Thus, these scanning techniques eliminate the need of mechanical displacements of the light sources or the samples. Nevertheless, these techniques are limited to generating arrays of light fields, lacking capability to independently manipulate positions of individual focal spots. Consequently, they usually fall short when come to controlling individual focal positions and generating focal light with customized structured modes at the scanning plane.

In this work, we present a novel principle for flexibly controlling subwavelength focusing and scanning, by using the unique Fourier translation property of a topology-preserved flat lens. Based on the shift theorem of the Fourier translation, a designed flat lens (the thickness is only 60 nm), together with the SLM, is able to achieve controllable shifting and scanning of the focal spot. Due to the high numerical aperture of the flat lens, the resultant full width at half maximum (FWHM) of the focal spot is at the subwavelength scale. We realize a scanning area of 10 μm × 10 μm, by adjusting the designed phase holograms. Moreover, the presented platform can also generate multifocal spots, with each focal spot independently controlled by the incident phase shift. Due to the topology-preserved property of flat lens, the proposed technique allows generation and manipulation of the focal light fields, hence, realizing spatially structured light including the vortex beam and the vector beam at the focal plane. Finally, we utilize our system in scanning imaging, demonstrating a spatial resolution up to 700 nm. In addition, the technique is also expected to be applied in other fields such as the laser lithography. Our demonstrations provide a promising avenue for subwavelength focusing control, which would stimulate continued investigations in the relevant applications.

## 2 Theory

**Figure 1:**
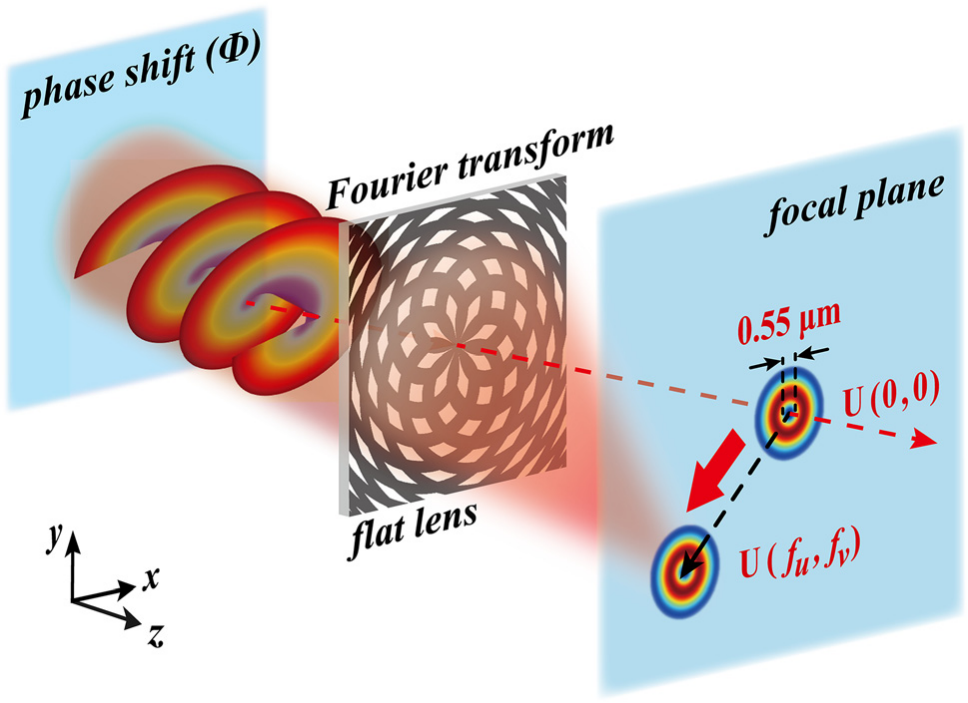
Schematic diagram of controlling subwavelength focusing. The flat lens with Fourier transform functionality transforms initial phase shift information into displacement of the focal point. Owing to the topology-preserved property of the flat lens, the method proposed can manipulate optical fields with arbitrary spatial structures, including scalar fields and vortex fields.

Two-dimensional Fourier transform is one of the most important characteristics of lens and is also a fundamental technique in optical information processing [49], [50]. Fourier transform operations are generally performed using complex and expensive electronic spectrum analyzers. In fact, this complex analog operation can be performed with light using more concise optical devices such as the optical lens. In this study, we consider using the Fourier transform of a particularly designed flat lens, realizing dynamic control of the focal light spots. The field distribution *U*(*u*, *v*) at the focal plane behind the flat lens can be expressed as:

(1) $$U\left(u,v\right)=\overset{+\infty }{\underset{-\infty }{\iint }}E\left(x,y\right)\exp\left[-i\frac{2\pi }{\lambda z_{f}}\left(xu+yv\right)\right]dxdy,$$

where *λ* denotes the wavelength, and *z*_*f*_ the expected focal length of the flat lens. (*u*, *v*) represent transverse coordinates in the focal plane, while (*x*, *y*) define the incident plane of light field *E*(*x*, *y*). The schematic diagram of the proposed mechanism for controlling the subwavelength focusing is illustrated in Figure 1. We introduce a phase term *ϕ* to control the focus spot displacement of the incident light *E*(*x*, *y*), which is defined as:

(2) $$\phi =\exp\left[-i\left(k_{x}x+k_{y}y\right)\right],$$

where *k*_*x*_ and *k*_*y*_ (their units are μm^−1^ in this paper) represent spatial frequencies that reflect phase changes of plane wave along the transverse plane. The light field incident on the flat lens is, therefore, expressed as *E*(*x*, *y*) ⋅ *ϕ*. From a physical point of view, the linear phase shift, which acts as a transverse momentum added to the incident light, would induce a steering angle. The distribution of light field at the back focal plane of the flat lens is given by

(3) $$\begin{array}{cc} & U\left(u+f_{u},v+f_{v}\right)\\ & \;=\overset{+\infty }{\underset{-\infty }{\iint }}E\left(x,y\right)\cdot \exp\left[-i\frac{2\pi }{\lambda z_{f}}x\left(u+\lambda z_{f}\frac{k_{x}}{2\pi }\right)\right]\\ & \;\cdot \exp\left[-i\frac{2\pi }{\lambda z_{f}}y\left(v+\lambda z_{f}\frac{k_{y}}{2\pi }\right)\right]dxdy, \end{array}$$

where *f*_*u*_ and *f*_*v*_ are the transverse displacements. Therefore, after adding the phase shift information to the incident light, the coordinate of the focus becomes $\left(u+f_{u},v+f_{v}\right)$ . Based on the shift theorem of the Fourier translation, these two displacement factors are determined by

(4) $$\begin{array}{cc} f_{u}=-\lambda z_{f}\frac{k_{x}}{2\pi }, & \\ f_{v}=-\lambda z_{f}\frac{k_{y}}{2\pi }. \end{array}$$

Clearly, if there is no phase shift (*k*_*x*_ = *k*_*y*_ = 0) added to the incident light, the focus coordinate (*u*, *v*) remains at the optical axis defined as (*u* = 0, *v* = 0) (Figure 1). After applying the phase shift information, the coordinate of the focal point becomes (*f*_*u*_, *f*_*v*_). Thus, we can arbitrarily control the transverse position of the focal spot by applying different values of *k*_*x*_ and *k*_*y*_.

## 3 Results

Using super-oscillatory lens/metalens for optical scanning facilitates the realization of integrated systems. However, these metasurface lenses are usually made of deep subwavelength resonant or nonresonant nanoscatters, which are very sensitive to the incident light parameters such as the polarization, phase, and wavelength. The sensitivity of the nanoscatters to the incident light greatly limits their performances or functionalities.

**Figure 2:**
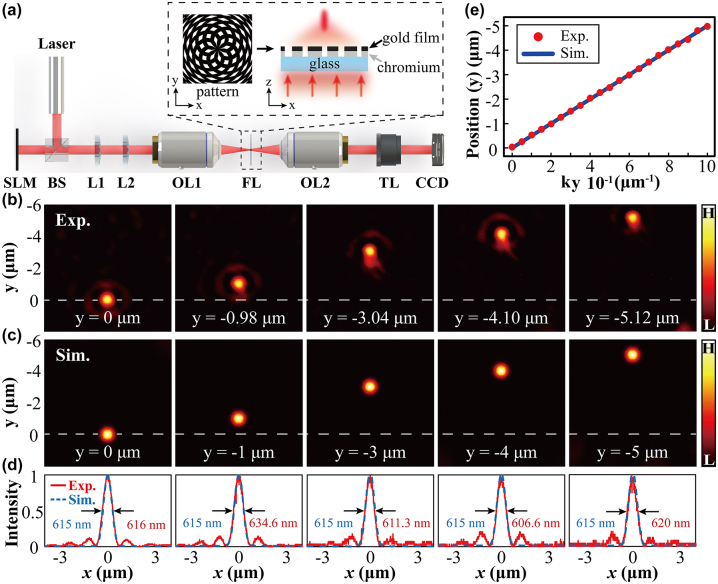
Experimental results of controlling the shift of the focal point in the *y*-direction. (a) Experimental setup. SLM, spatial light modulator; BS, beam splitter; L1, L2, lens; OL1, object lens (10×); FL, flat lens; OL2, object lens (150×); TL, tube lens; CCD, charge-coupled device. Inset: The layout of the designed flat lens (top view and side view). (b) Experimental and (c) simulation results of focus shift in the *y*-axis direction from *y* = 0 μm to *y* = −5 μm. (d) The cross sections of the normalized intensity profiles of the focal light fields along *x*-axis. The red curves denote the experiment (Exp.), while the blue dish curves represent the simulation (Sim.). (e) Relation between phase shift and displacement. The positions of the center of focus point change with *k*_*y*_, when setting *k*_*x*_ = 0 μm^−1^ and *k*_*y*_ varying from 0 μm^−1^ to 1 μm^−1^. The red data points are the experimental results (Exp.), and the blue line is the theoretical simulation results (Sim.).

To overcome this problem, we design and fabricate a topology-preserved flat lens (the thickness is 60 nm), according to the technique reported in [51]. Theoretically, the structure of the flat lens relies on an amplitude-only hologram resulted from interference between an angular cosine wave and a spherical wave. While the angular wave features a rotationally symmetric flat lens pattern that guarantees the topology-preserved property, the spherical wave encodes the lens’s information, enabling the Fourier transform. We search for an encoding technique to binarily truncate the interference pattern [51]. The resultant layout of the designed flat lens is shown in the inset of Figure 2(a). It is comprised of spatially shaped apertures, featuring information of the Fourier transform lens. The white section of the structure fully transmits the beam, while the black section completely blocks it. To fabricate the flat lens, at the beginning, a chromium film (being an adhesion layer) with a thickness of 10 nm and a gold film with a thickness of 50 nm are deposited successively onto a glass substrate, by using the physical vapor deposition technique. Then, a positive photoresist whose thickness is 1.2 μm is spin-coated onto the gold film and baked for 2 min at the temperature of 100 °C. We mention that continuous baking can gradually evaporate photoresist’s solvent and enhance the viscosity of the photoresist. Thirdly, a designed mask obtained from the binary modulation technique is carefully aligned and placed onto the photoresist. An ultraviolet (UV) laser source is applied to illuminate the sample for about half minute and develop for about 1 min. After UV exposure, the mask’s pattern is transferred to the photoresist. Finally, we consider using an ion beam to etch and peel off the metallic films outside the mask’s pattern. The obtained sample after ion-beam etching is put into an acetone solution, dissolving the photoresist. The final pattern is the desired flat lens, as shown in Figure 2(a). Note that the fabricated flat lens has a numerical aperture (NA) of 0.7 and a focal length of *z*_*f*_ = 50 μm, enabling focusing light into the subwavelength scale.

Even though the designing mechanism of the flat lens has been demonstrated in detail, flexibly controlling subwavelength focusing and transverse scanning is not achieved. Here, we demonstrate a new computational scanning technique that is based on the Fourier transform property of the flat lens. This flat lens property allows for the conversion of an initial phase shift of light into a spatial displacement of its subwavelength focus. Importantly, we develop a technique that involves dynamic loading of a series of phase profiles onto the SLM to achieve scanning of an image.

The SLM allows to dynamically load the phase maps, enabling realization of the intricate beam patterns [52]. In experiment, we consider using the encoding technique proposed by Bolduc [53], [54] to generate the desired phase-only mask [see Eq. (2)]. Figure 2(a) illustrates an experimental setup to implement our idea. The laser (*λ* = 632.8 nm) is linearly polarized and carefully expanded and collimated by using a beam expander (not shown in the Figure 2). It is then divided by a beam splitter (BS). The split beam is normally injected onto a reflective phase-only SLM (Holoeye LETO II, 1,920 × 1,080, pixel size 6.4 μm). The phase-modulated beam, after propagating through a telescopic system, is focused by the flat lens. We use an objective (OB) lens together with a tube lens (TL) to image the focal light field onto a charge-coupled device (CCD) with a pixel size of 1.4 μm.

Firstly, we demonstrate subwavelength focusing shifting along one-dimensional (1D) direction. Figure 2(b) demonstrates experimental results, showing controllable shifting of the focal point along the *y*-axis at the focal plane. Without phase modulating, the focus is located at the origin point (the optical axis *y* = 0). By setting the *k*_*y*_ (unit: μm^−1^) values as 0, 0.2, 0.6, 0.8, and 1, we obtain the focus positions located at *y* = 0 μm, −0.98 μm, −3.04 μm, −4.1 μm, and −5.12 μm, respectively. It is evident from Figure 2(b) that, when increasing the value of *k*_*y*_, the focal spot gradually moves away from the baseline (dashed line).

We perform simulations to confirm our experimental measurements. The light field after being phase modulated and passing through the flat lens is simulated according to the following paraxial Helmholtz equation

(5) $$i\frac{\partial E^{\prime }}{\partial z}+\frac{1}{2k}\left(\frac{\partial^{2}E^{\prime }}{\partial x^{2}}+\frac{\partial^{2}E^{\prime }}{\partial y^{2}}\right)=0$$

where *E*′ denotes the transmission field at a distance of *z*, and *k* = 2*π*/*λ* is the free-space wavenumber. Here, we define *z* = 0 as a plane where the flat lens is placed. The transmission light field at the distance of *z* > 0 is considered. We solve the paraxial wave equation by means of the fast-Fourier transform algorithm, with initial condition *E*′(*x*, *y*, *z* = 0) = *E*(*x*, *y*) ⋅ *ϕ* ⋅ *t*(*x*, *y*), where *E*(*x*, *y*) is the incident light filed, and *t*(*x*, *y*) is the transmission function of the flat lens. To match the experimental parameters, we set *λ* = 632.8 nm and *z*_*f*_ = 50 μm. Under these settings, we perform numerical simulations, with simulated outcomes displayed in Figure 2(c). The simulations show a good agreement with the experimental measurements, as judged from the intensity distributions in Figure 2(c) and their profiles in Figure 2(d). The simulated outcome indicates the FWHM about 615 nm, which matches approximately the experiment. The relationship between phase modulating frequency (*k*_*y*_) and focal displacement (*y*) is illustrated in Figure 2(e). The red data points denote the experiments, while the blue line indicates the simulation. We observe a linear correlation between them, which can be described by a linear function *y* = −5 μm^2^ ⋅ *k*_*y*_. It is found that these results also agree well with the corresponding theoretical model in Eq. (4). According to it, we calculate the slope as −5 μm^2^, which is consistent with the relationship shown in Figure 2(e). Within these results, we can precisely control motion of the focus, enabling potential application in various fields such as scanning imaging (as discussed below).

**Figure 3:**
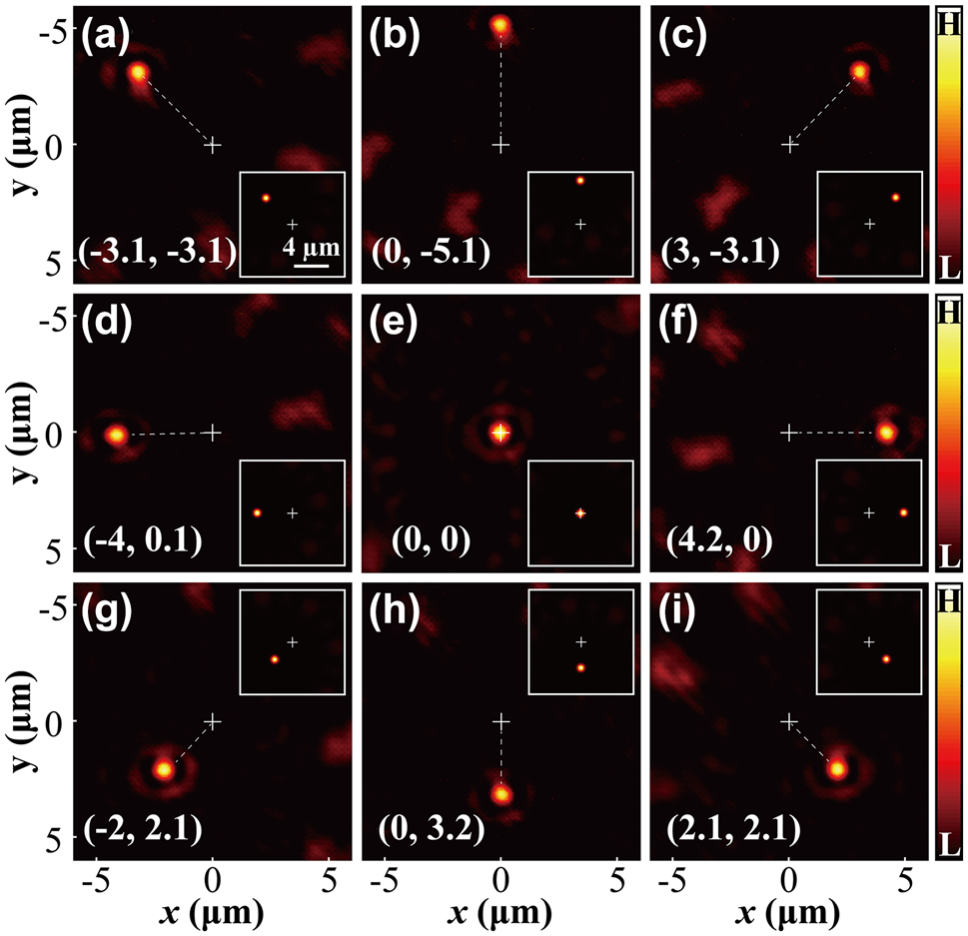
Results of controlling the focal shift along eight different directions. The experimentally measured light fields at the focal plane are presented in (a–i). The focus coordinates (*f*_*u*_, *f*_*v*_) are placed at the bottom of each panel. Unit: μm. The corresponding theoretically calculated distributions are shown in the insets of these panels for the purpose of comparison. The insets share identical scale, with scale bar 4 μm.

In the following, we demonstrate subwavelength focusing shifting in two-dimensional (2D) direction. According to the Fourier translation property of the flat lens, the introduction of a phase shift characterized by *k*_*x*_ and *k*_*y*_ results in a focal displacement featured by *f*_*x*_ and *f*_*y*_, respectively. We demonstrate this 2D controllable shifting, with both the experimental and simulated results shown in Figure 3. Again, when the incident light is not modulated, the focus of the flat lens is located at the optical axis position, as shown in Figure 3(e). The other panels in Figure 3 demonstrate that we are able to shift the focus along eight directions by using different combinations of the parameters *k*_*x*_ and *k*_*y*_. We should note that, theoretically we can obtain a sufficiently large focal displacement, while, experimentally, due to the limited pixel size of the SLM, we can only achieve a maximum focal shifting about 5 μm, resulting in a scanning area about 10 μm × 10 μm. The experimental measurements match well with the simulations. Due to limited fabrication precision, the fabricated flat lens is not perfectly consistent with the design pattern, leading to slight crosstalk between different diffractive wave vectors and, hence, causing inevitable interference noise.

We demonstrate feasibility of effectively realizing and controlling multifocal spots with the Fourier translation property. We note that the required amplitude and phase information for realizing the multifocus arrays is not expressed by the binary amplitude-modulated flat lens. We achieve this purpose by adding individual phase shifts to the incident light beam using the SLM. As an example, we show how to generate arbitrary multifocal spots having the Gaussian profile. This can be realized by adding multiple phase terms to the incident light field. We, therefore, rewrite Eq. (2) as:

(6) $$\phi_{n}=\exp\left[-\left(ik_{xn}x+ik_{yn}y\right)\right],$$

where *k*_*xn*_ and *k*_*yn*_ denote spatial frequencies that are used to modulate the *n*th focal spot. According to the diffraction theory in Eq. (3), we obtain the resultant light field distribution, which is composed of multifocal spots. The solution is expressed as:

(7) $$\begin{array}{c} E\left(f_{u1},f_{v1}\right)+E\left(f_{u2},f_{v2}\right)+\cdots +E\left(f_{un},f_{vn}\right)\\ =F\left\{E\left(x,y\right)\left[\phi_{1}+\phi_{2}+\cdots +\phi_{n}\right]\right\}, \end{array}$$

where F(⋅) denotes the Fourier transform. As described by Eq. (7), the superposition of the individual phase modulation leads to a light field distribution, which is composed of multiple focal spots. Their focal positions are only dependent on the respective phase modulation function *ϕ*_*n*_. Therefore, the position of each focus can be controlled independently.

**Figure 4:**
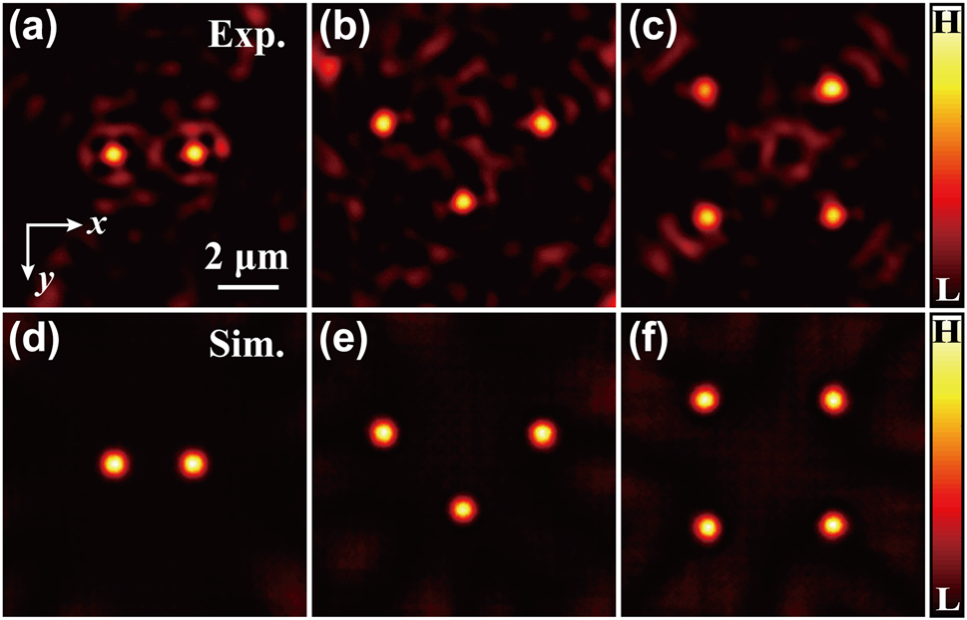
Results for generation and manipulation of the multifocal spots. (a, d) Two-focus array: the coordinates of the two focal points with respect to the origin are [(−1.3, 0), (1.35, 0)] for (a) and [(−1.3, 0), (1.3, 0)] for (d). (b, e) Triangular array (triple-focus array): the coordinates of the three points are [(−2.6, −1), (2.63, −1), (0, 1.5)] for (b) and [(−2.6, −1), (2.6, −1), (0, 1.5)] for (e). (c, f) Four-focus array: the coordinates of the four points are [(−2.2, −2.12), (1.98, −2.16), (−2.12, 2.1), (2, 2)] for (c) and [(−2.2, −2.1), (2, −2.1), (−2.1, 2.1), (2, 2)] for (f). The unit of coordinates is μm. The (a), (b), and (c) are the experiment results, while the (d), (e), and (f) are the simulation results corresponding to (a), (b), and (c), respectively. Exp.: Experiment result; Sim.: Simulation result. All panels share the same scale, with scale bar 2 μm.

Figure 4 shows an example of flexibly controlling individual spots in the multifocal array. At the beginning, we introduce the phase shift information for the two focal spots, written as

(8) $$\phi_{1}+\phi_{2}=\exp\left(-ik_{x1}x\right)+\exp\left(-ik_{x2}x\right),$$

where *k*_*x*1_ = 0.25 μm^−1^ and *k*_*x*2_ = −0.25 μm^−1^. We observe clearly that the original one focal spot located at the optical axis (0, 0) split into two focal spots, located at (−1.3, 0) and (1.35, 0) (the unit is μm), respectively, as shown in Figure 4(a). Then using the same procedure, we demonstrate generations of triangular array (triple-focus array) and four-focus array, with experimental results shown in Figure 4(b) and (c), respectively. We repeat these results numerically, by solving the paraxial wave equation, with outcomes shown in Figure 4(d)–(f). Our simulations are in accordance with the experiments. Figure 4 shows that the individual focal spots share approximately identical Gaussian width (about 615 nm). We can simultaneously control the movement of each focal spot, by individually encoding their phase information into the incident light. In the multifocal experiments, we observe stronger interference noise than the single-focal experiments. This is because the introduction of the multifocal spots (a superposition of diffractive waves with different phase information) leads to the superposition of interference noise coming from individual focal light fields (see Figure 3 for comparison).

**Figure 5:**
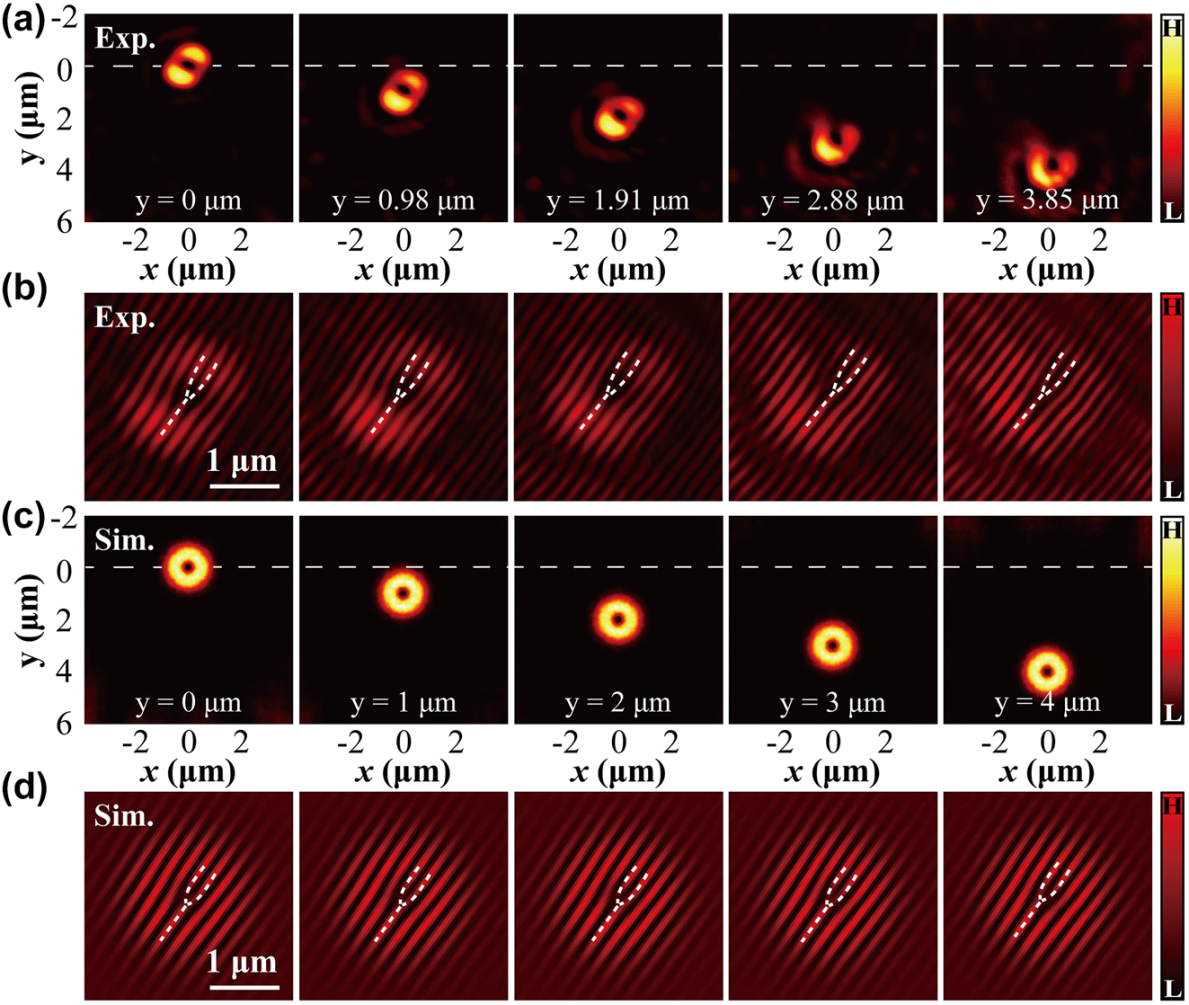
Controlling the focal shifting of the vortex beam: (a) the experimental focus displacements varying with different phase modulation frequency: *k*_*y*_ = 0, −0.2, −0.4, −0.6, and −0.8 (unit: μm^−1^). (c) The corresponding simulation result to (a). (b, d) The corresponding plane-wave interference fringes for measuring vortex phase: (b) experiment and (d) simulation. Panels in (b, d) share the same scale, with scale bar 1 μm.

In addition to the generation and manipulation of the subwavelength focal spots having the Gaussian mode, our technique is also suitable for subwavelength control of the focal spots having the higher-order light modes such as the Laguerre–Gauss vortex modes. This is indicated by the incident light field *E*(*x*, *y*) in Eq. (3), which is not limited to the cases discussed above. It represents arbitrary light forms including scalarly and vectorially structured light [55]–[57]. One implementation of the structured light, i.e., the vortex beam [58], is considered in the experiments. As mentioned, the flat lens does not alter the incident vortexing phase of the LG beams. Similar to the previous demonstrations, we generate different phase holograms for controlling the focal vortex beam by using the SLM. In the experimental setup, we introduce another optical path to interfere with the focal vortex spot, measuring its phase. As illustrations, we set *k*_*x*_ = 0 μm^−1^ and vary *k*_*y*_ from 0 μm^−1^ to −0.8 μm^−1^, with an interval of −0.2 μm^−1^. The corresponding experimental results are presented in Figure 5(a). The positions of the focused vortex light beam are obtained as *y* = 0 μm, 0.98 μm, 1.91 μm, 2.88 μm, and 3.85 μm, respectively. It is relevant to mention that, as the value of *k*_*y*_ changes from 0 μm^−1^ to −0.8 μm^−1^, the focal vortex spot moves from *y* = 0 μm to *y* = 3.85 μm. In simulations, we reproduce the outcomes for the controllable vortex light focusing. We replace the initial condition in Eq. (5) by considering the following light mode:

(9) E(r,z=0)=A(r)exp(iθ),

where $A\left(r\right)=r/\sigma_{0}\exp\left(-r^{2}/\sigma_{0}^{2}\right)$ represents the LG function and *θ* denotes an azimuthal angle defined as *θ* = arctan(*y*/*x*) with value ranging between −*π* and *π*. Here, *σ*_0_ denotes the Gaussian width and $r={\left(x^{2}+y^{2}\right)}^{1/2}$ . In this scenario, we repeat the focusing phenomenon of the vortex beam by solving the paraxial Helmholtz equation above, with numerical results shown in Figure 5(c). Theoretically, the focal displacements corresponding to *k*_*y*_ are obtained as follows: *y* = 0 μm (*k*_*y*_ = 0 μm^−1^), *y* = 1 μm (*k*_*y*_ = −0.2 μm^−1^), *y* = 2 μm (*k*_*y*_ = −0.4 μm^−1^), *y* = 3 μm (*k*_*y*_ = −0.6 μm^−1^), and *y* = 4 μm (*k*_*y*_ = −0.8 μm^−1^). It is seen that both the experimental and theoretical focal fields exhibit a donut-shaped distribution, with peak-to-peak value measured as ≈1.1μm , and the FWHM value of the central dark hole measured as ≈0.55μm . Figure 5(b) and (d) shows, respectively, the experimental and simulated plane-wave interference fringes for characterizing the vortex phase. From these results, it is evident from the dislocations that the topological phase can be maintained after the modulated structured light passing through the flat lens.

**Figure 6:**
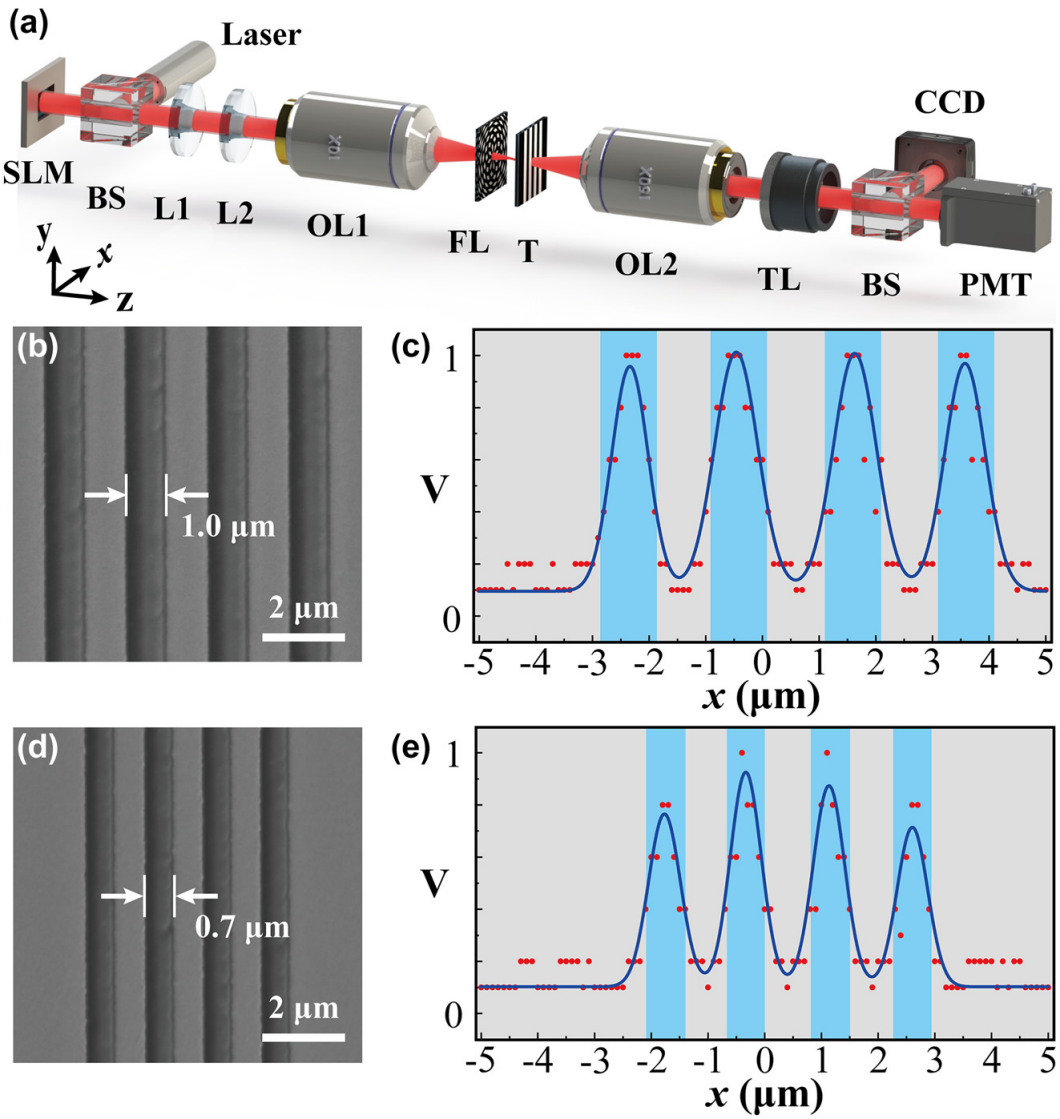
The experimental result of scanning image. (a) Schematic of the optical system for scanning imaging. SLM, spatial light modulator; BS, beam splitter; L1, L2, lens; OL1, object lens (10×); FL, flat lens; T, target; OL2, object lens (150×); TL, tube lens; CCD, charge-coupled device; PMT, photomultiplier tube. (b, d) SEM images of the design targets. The slit width is (b) 1 μm and (d) 700 nm. (b) and (d) share the same scale, with scale bar being 2 μm. The spacing between two neighboring slits are (b) 1 μm and (d) 700 nm. For objects (b) and (d), the corresponding scanning imaging results are (c) and (e), respectively. The blue rectangular slits indicate the positions of the targets. The red points are the experimental data, and the blue lines are the fitted curves.

Finally, we are interested in the application of such technique in the optical scanning imaging. To this end, we modify our experimental system, as depicted in Figure 6(a). The imaging target consists of four side-by-side rectangular seams. Each rectangular slit has a width of 1 μm and the spacing between adjacent slits is 1 μm. The imaging target is placed at the back focal plane of the flat lens. Figure 6(b) illustrates SEM image of the fabricated 1D slit array. To resolve this structure, we dynamically control the movement of a single subwavelength focal spot, realizing 1D scanning of the slit structure. A photomultiplier tube (PMT) is used to collect intensity of light after passing through the slit array. The obtained signal represented by electrical voltage is displayed on an oscilloscope. According to Eq. (4), we set *k*_*y*_ = 0 μm^−1^ and increase the value of *k*_*x*_ from 0 μm^−1^, within an interval of *k*_*x*_ = 0.02 μm^−1^. This results in a scanning step size of 100 nm. We generate these phase-only holograms and dynamically load them onto the SLM. While the focal point is gradually moved along the slit array, we collect the voltage as a function of the displacement, with result shown in Figure 6(c). The red points are the recorded experimental data, while the blue curve is the fitting line using the Gaussian function based on the experimental data. In order to see the slit array pattern, Figure 6(c) also includes four blue identical rectangles showing the positions of the slits. From the experimental results, we obtain four Gaussian peak values, indicating specific positions of the slits. The signal variation in Figure 6(c) is a direct indication of the 1D slit array. Furthermore, we utilize this system to resolve the slits with a smaller slit width (700 nm). Figure 6(d) shows the SEM image, while Figure 6(e) depicts the corresponding scanning result. We still observe the clear Gaussian peaks, corresponding to the signals of the slit aperture. We note that the peak values of the four Gaussian curves exhibit differences. We attribute this to the relatively large scanning step size. In this case, the FWHM of the focal spot is about 615 nm, while the slit width is comparable to the spot size. At this level, a relatively large scanning step size would lead to the fact that the focal spot is most likely not located at the slit center, but at the different edge positions of the slit. As a result, the recorded signal by the PMT is different when we are scanning through the different slits. Such intensity difference would lead to a resolved optical image with inhomogenous intensity distribution, which, to some extent, reduces the spatial resolution. Note that the spatial resolution can be improved further when using the higher-numerical-aperture flat lens. We mention that, the SLM has a refresh rate of 60 Hz, allowing us to load 60 phase holograms per second. Thus, our system presented here is capable of realizing high-speed subwavelength scanning.

## 4 Conclusions

In summary, we believe that our demonstration provides a clear example of how the Fourier translation can be utilized to control the subwavelength focusing and scanning. Specifically, a designed computer-generated phase-only hologram is loaded onto the SLM, resulting in a subwavelength focal shift in transverse plane. Based on this principle, we have experimentally and theoretically demonstrated subwavelength focusing and scanning both in one and two dimensions, by properly tailoring the modulation frequencies *k*_*x*_ and *k*_*y*_ of the holograms. Interestingly, a controllable multifocal light field is also realized by adding individual phase terms to the incident light. The presented flat lens exhibits the topology-preserved property, enabling generation and control of the focused structured light at the subwavelength scale, which cannot be achieved via conventional high-numerical-aperture Fourier lens because of its significant depolarization effect [59]. We have further demonstrated application of this technique in computational scanning imaging, achieving a scanning area about 10 μm × 10 μm, and a spatial resolution up to 700 nm. It is worth mentioning that the presented flat lens becomes a promising element in the area of frontier integrated optics since the thickness of the flat lens is only 60 nm. The inclusion of the flat lens in the scanning imaging system make it promising in a wide range of potential applications, which require precise control of subwavelength focusing and scanning such as optical manipulation, lithography, and information transfer, not limited to the scanning imaging.
